# Supplementary material for: A candidate sex determination locus in amphibians which evolved by structural variation between X- and Y-chromosomes
Source: Nat Commun. 2024 Jun 5;15:4781. doi: 10.1038/s41467-024-49025-2 (PMC11153619; doi:10.1038/s41467-024-49025-2)
Supplement: Supplementary file 1 — Supplementary Information [file 41467_2024_49025_MOESM1_ESM.pdf]

Supplementary information for

A candidate sex determination locus in amphibians which evolved by structural variation between X- and Y-chromosomes

Heiner Kuhl[1,*], Wen Hui Tan [2,*], Christophe Klopp[3], Wibke Kleiner[1], Baturalp Koyun[1,4], Mitica Ciorpac[5,6], Romain Feron[7,8], Martin Knytl[9,10], Werner Kloas[1], Manfred Schartl[11,12], Christoph Winkler[2,§], Matthias Stöck[1,§]

[1] Leibniz-Institute of Freshwater Ecology and Inland Fisheries, IGB, Müggelseedamm 301 & 310, 12587 Berlin, Germany,
[2] Department of Biological Sciences and Centre for Bioimaging Sciences, National University of Singapore, 14 Science Drive 4, Block S1A, Level 6, Singapore 117543,
[3] SIGENAE, Plate-forme Bio-informatique Genotoul, Mathématiques et Informatique Appliquées de Toulouse, INRAe, 31326 Castanet-Tolosan, France
[4] Bilkent University, Department of Molecular Biology and Genetics, Faculty of Science, SB Building, Ankara, Turkey 06800,
[5] Danube Delta National Institute for Research and Development, Tulcea 820112, Romania,
[6] Advanced Research and Development Center for Experimental Medicine – CEMEX, "Grigore T. Popa", University of Medicine and Pharmacy, Mihail Kogălniceanu Street 9-13, Iasi 700259, Romania,
[7] Department of Ecology and Evolution, University of Lausanne, Lausanne, Switzerland,
[8] Swiss Institute of Bioinformatics, Lausanne, Switzerland,
[9] Department of Cell Biology, Faculty of Science, Charles University, Viničná 7, Prague, 12843, Czech Republic
[10] Department of Biology, McMaster University, 1280 Main Street West, Hamilton L8S 4K1, Ontario, Canada
[11] Developmental Biochemistry, Biocenter, University of Wuerzburg, Am Hubland, 97074 Wuerzburg, Germany,
[12] The Xiphophorus Genetic Stock Center, Department of Chemistry and Biochemistry, Texas State University, San Marcos, Texas, TX, 78666, USA.

[*] These authors contributed equally to the paper.

[§] Co-corresponding authors: cwinkler@nus.edu.sg ; matthias.stoeck@igb-berlin.de

---

Supplementary Figures 1-11

Supplementary Text 1

Legends of Supplementary Figures

Supplementary Figure 1: **Analyses of ddRAD markers in three genetic families of *B. viridis* using RADsex[81].** Of the 443,378 markers present in at least one individual with a coverage >5; 96 markers were significantly associated with male sex ($p < 0.05$ after Bonferroni correction), but none of these markers were found in all males.

Supplementary Figure 2: **Alignment of ddRAD markers shown in Supplementary Figure 1 to the *B. viridis* reference genome.** 64 of the 96 markers significantly associated with sex were located on chromosome 1 (scaffold 1, scf1), one marker was aligned to chromosome 7 (scf7), five markers were aligned to unplaced scaffolds, and 26 markers were not aligned uniquely or with a mapping quality lower than 20.

Supplementary Figure 3: **Mapping pool-sex short reads to the *B. viridis* reference genome with SNP-heterozygosity in males (a) *vs*. females (b) on scaffold (scf) 1.**

Supplementary Figure 4: Male-specific SNPs of six species over *bod1l* region. **a** SNPs show different blocks of male-specific heterozygosity between species, which exclude coding changes of *bod1l* as sex-determination mechanism. **b** Highest congruence of male-specific SNPs among all species is in the PCR marker/ ncRNA-region (red ellipsis). **c** Phylogenetic tree of haplotypes from the marked region shows clustering by gametologue. **d** sequences from the upstream region were clustered by species.

Supplementary Figure 5: Coverage distributions of X/Y haplotypes based on female/male pool-seq in *B. viridis*. a Y-haplotype, **b** X-haplotype.

Supplementary Figure 6: Female/male pool-seq coverage over haplotypes in *B. viridis*. a female pool-sequencing, **b** male pool-sequencing.

Supplementary Figure 7: X- and Y-haplotype comparisons by dot plots in the *bod1l*-region.

a X-*versus*-Y dot plot, **b** Y-*versus*-Y, **c** X-*versus*-X.

Supplementary Figure 8: Comparison of additive sex-specific expression of bod1l and ncRNA-Y and haplotype-specific expression in males. Bars represent mean values and error bars represent standard deviation.

Supplementary Figure 9: a, Y- and thus male-specific expression of the ncRNA-Y (204 bp PCR-product from cDNA) in larval green toads. PCR-products from cDNA (204 bp), obtained with primers NcRNAY_Ex1to2_F/_R; to the males(left), females(right); each sex is shown from Gosner stage 23-34 (10 days after fertilization) until early metamorphosis (Gosner 43-44) and one adult gonad for each sex; in females, only non-specific products occurred, since X-copies of *bod1l* do not contain and thus do not express this ncRNA. **b,** Gel image of a control-PCR using the females from (a).

Supplementary Figure 10: Enhancer prediction using the AI-based software i-enhance. Signal bars reaching from 0 to 2, predicting strong enhancer properties of the Y-specific 5'-end of *bod1l*, including a G-quadruplex-rich region on the non-template strand, overlapping with the ncRNA-Y.

Supplementary Figure 11: *Vasa*, *bod1l* expression and H3K4me3-methylation in *B. viridis* gonads, overview images and additional representative examples.
a-m, v*asa* and *bod1L* expression shown by RNA *in-situ* hybridization (ISH) on 20 µm transverse cryosections of female (**a, b, g, h**) and male (**c, d, f, i-m**) gonads, Gosner 36-37. DAPI was used to stain nuclei. Sense controls for RNA ISH are shown in **a, c, e**, **g**, right panels. Red boxes in **a, c, g, i** (left panel) indicate zoomed images shown in **b, d, h, k,** respectively. Yellow boxes in **b, d, h, k** (right panel) indicate zoomed images shown in **Fig. 5a-b,** respectively. Red boxes in l**, m** (left panel) indicate zoomed images shown in right panels of l**, m**. *Bod1L* staining on three different cryosections of male gonads are shown in k**, l, m**. Magenta asterisks indicate nuclei of germ cells; magenta arrows: cytoplasm of germ cells; yellow arrows: somatic cells not stained by RNA ISH; green arrows: somatic cells stained by RNA ISH. **n, o,** H3K4me3 and DAPI staining on 20 µm transverse cryosections of female (**l**) and male (**m**) *B. viridis* Bidder's organ and gonads, Gosner stage 43-44. Yellow boxes in **l, m** indicate zoomed images shown in **Fig. 5g**, **h,** respectively. White asterisks indicate H3K4me3-positive germ cell nuclei; white arrowheads, H3K4me3-positive somatic cell nuclei; yellow arrowheads, H3K4me3-negative somatic cell nuclei. Scale bars: (a, c, g, i) 50 µm, (b, d-f, h, k-o) 20 µm.

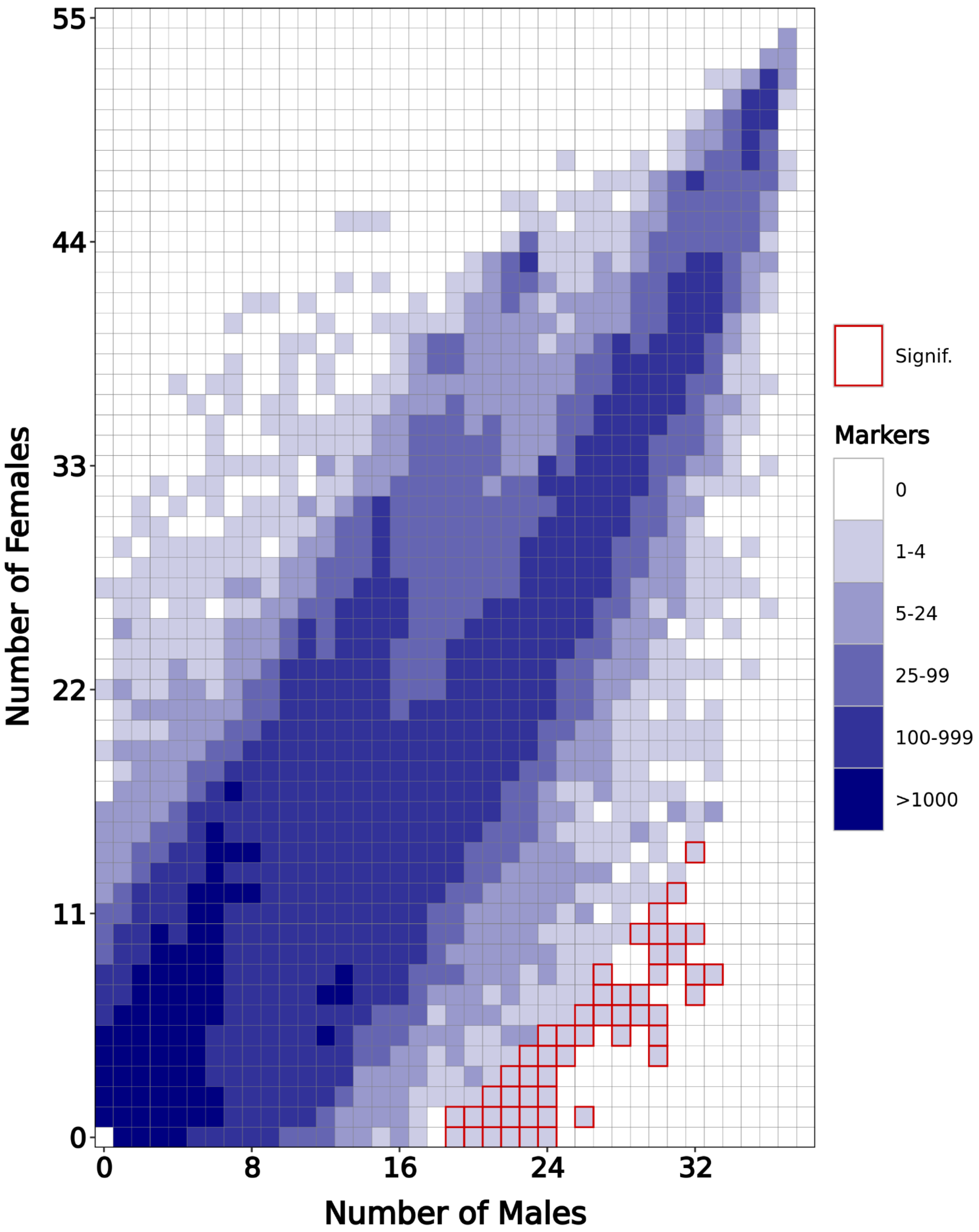

Supplementary Figure 1

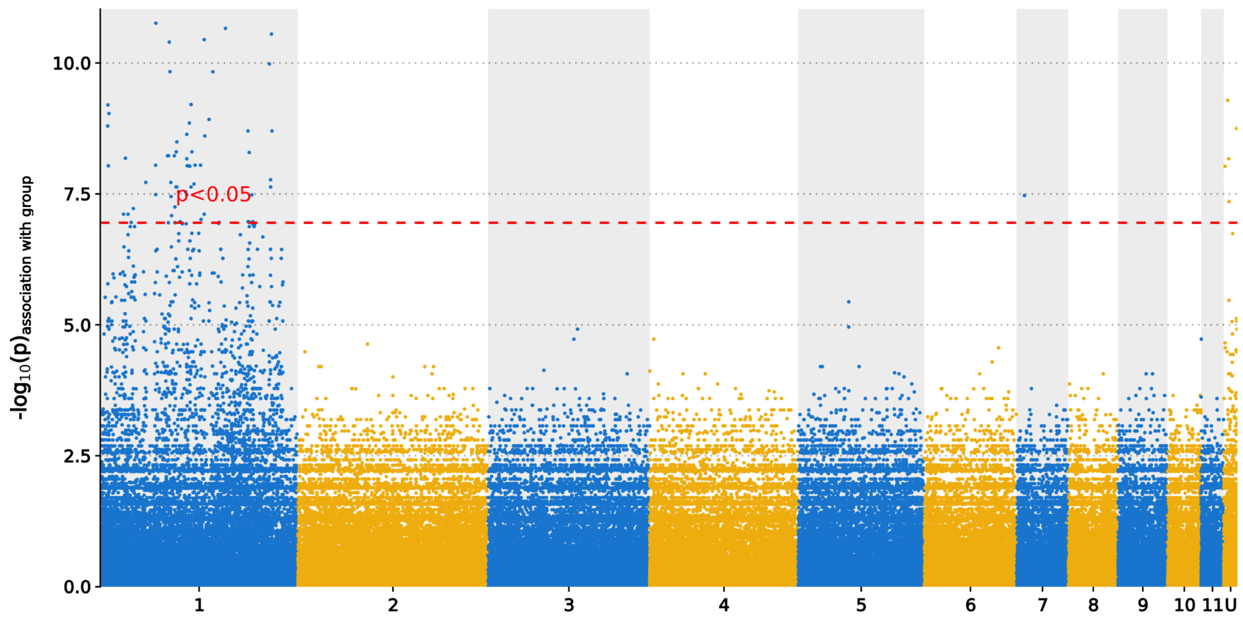

Supplementry Figure 2

Supplementary Figure 3

a

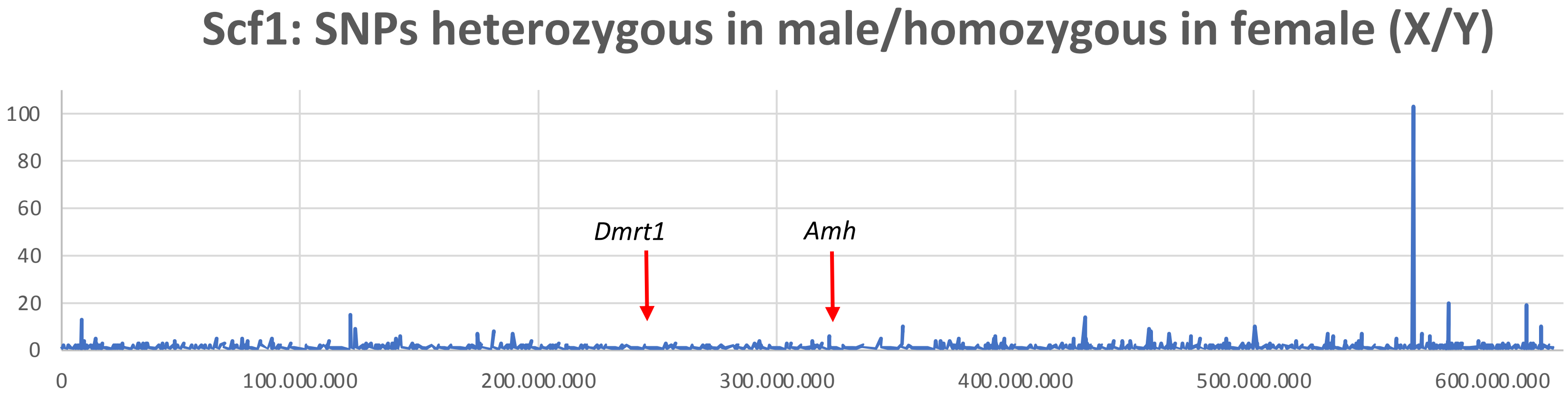

b

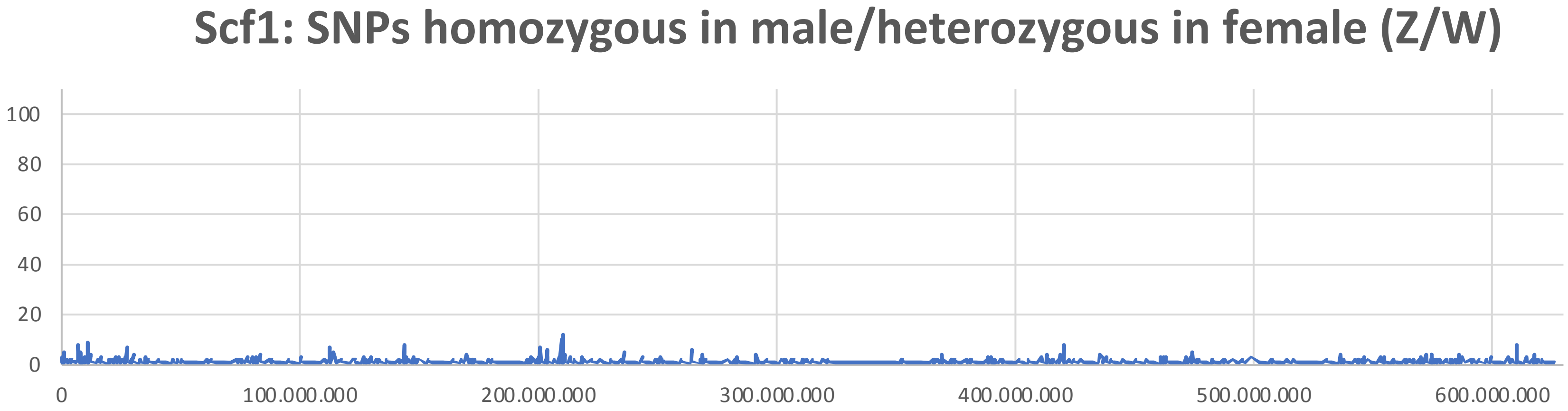

Supplementary Figure 4

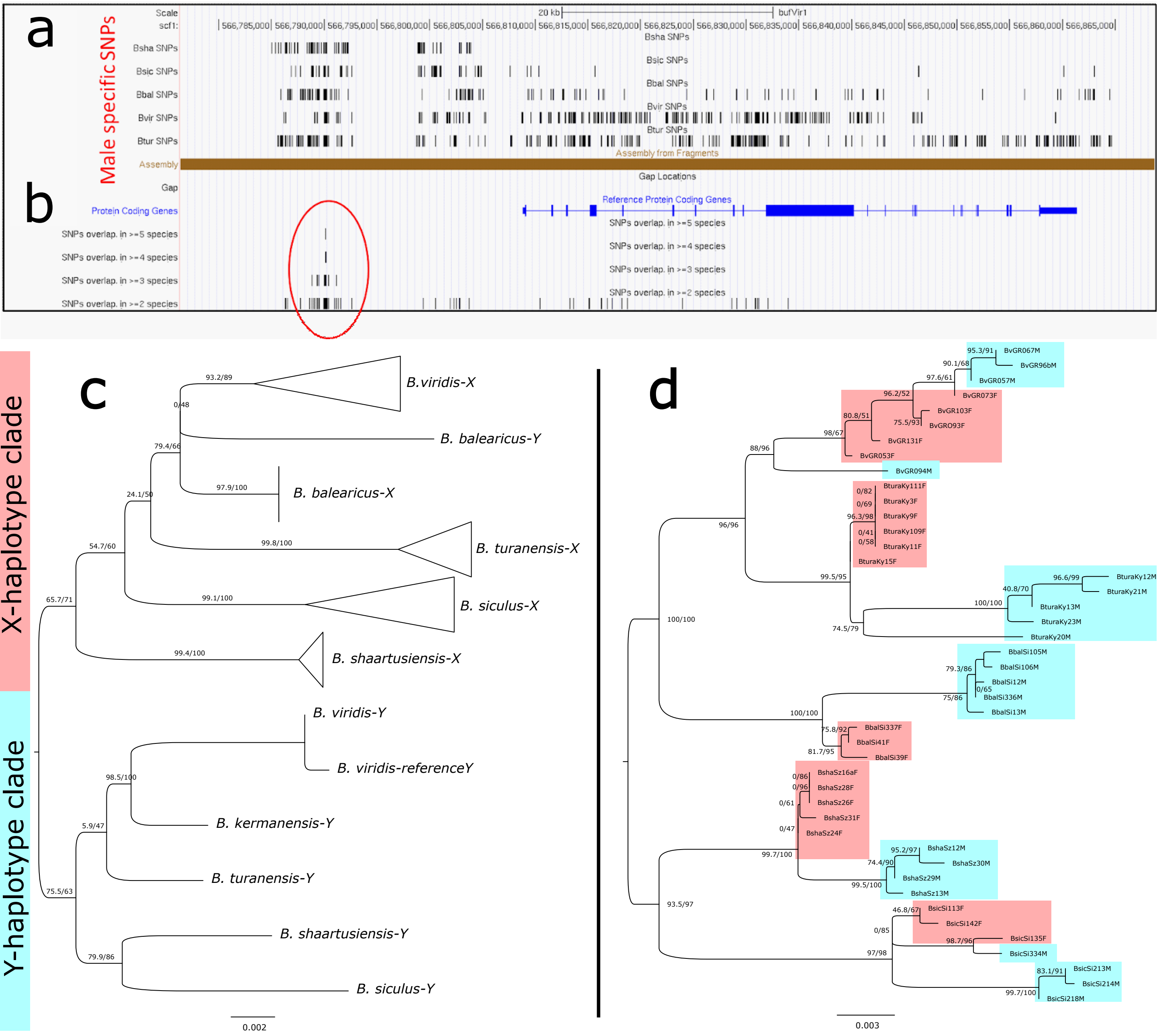

Supplementary Figure 5: Coverage distributions of X/Y haplotypes depending on female/male poolseq in *B. viridis*:

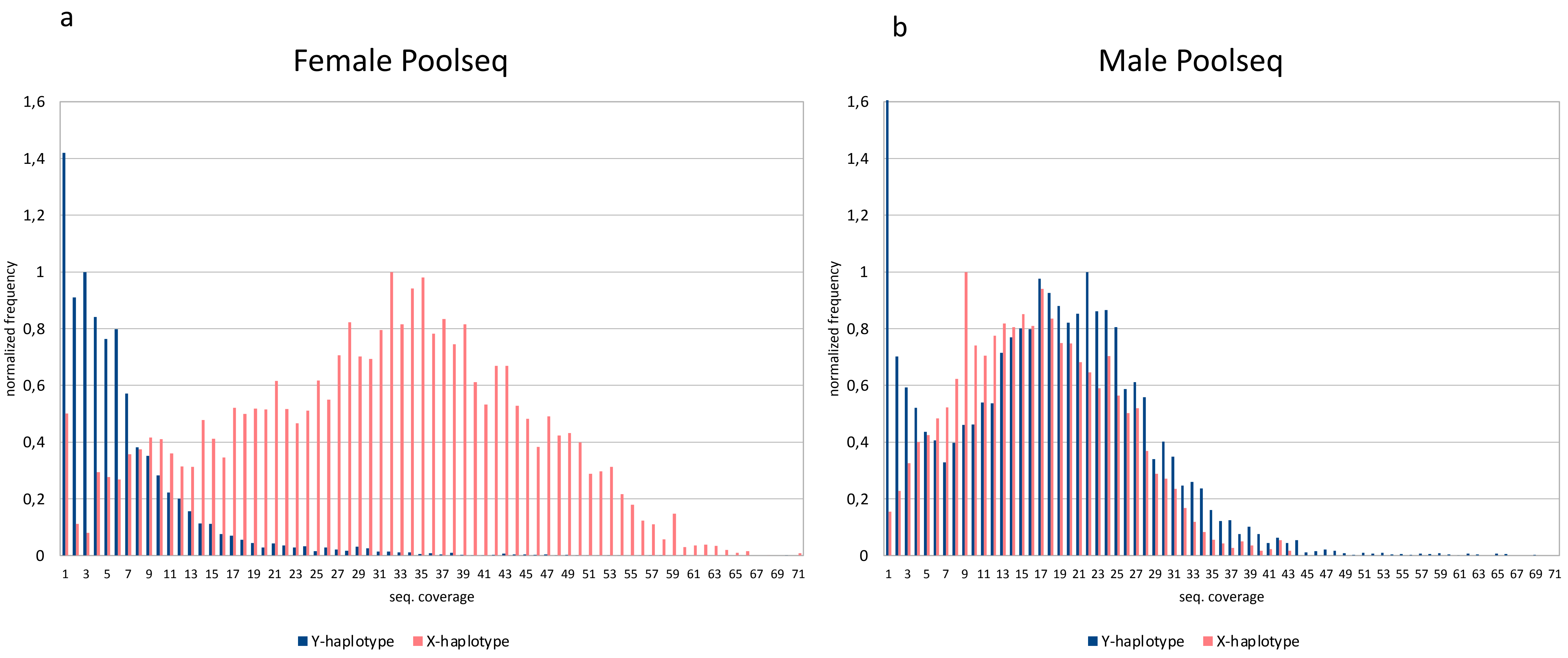

Supplementary Figure 6: Female/male poolseq coverage over haplotypes in *B. viridis*

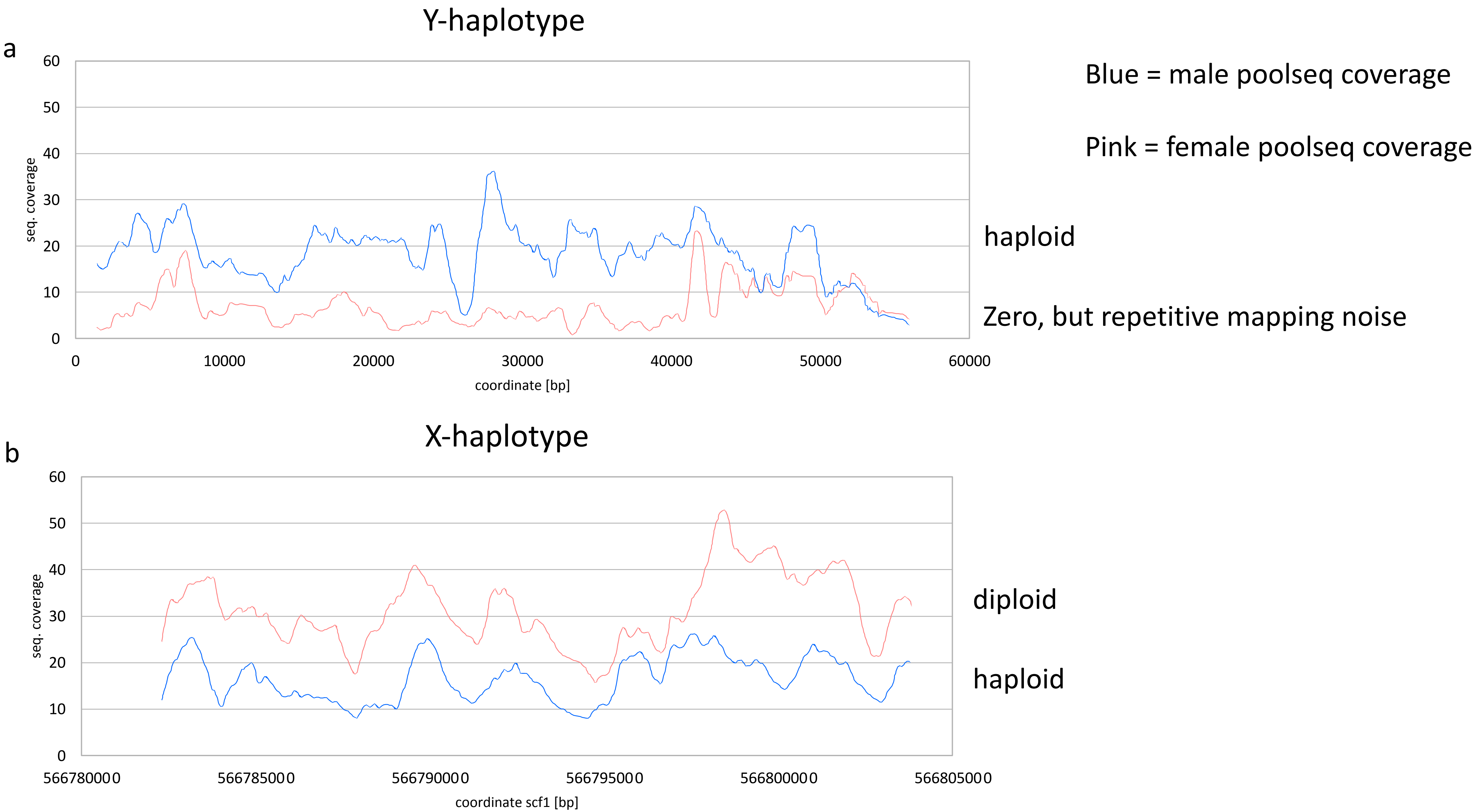

Supplementary Figure 7: X- and Y-haplotype comparisons by dot plots in the *Bod1L*-region

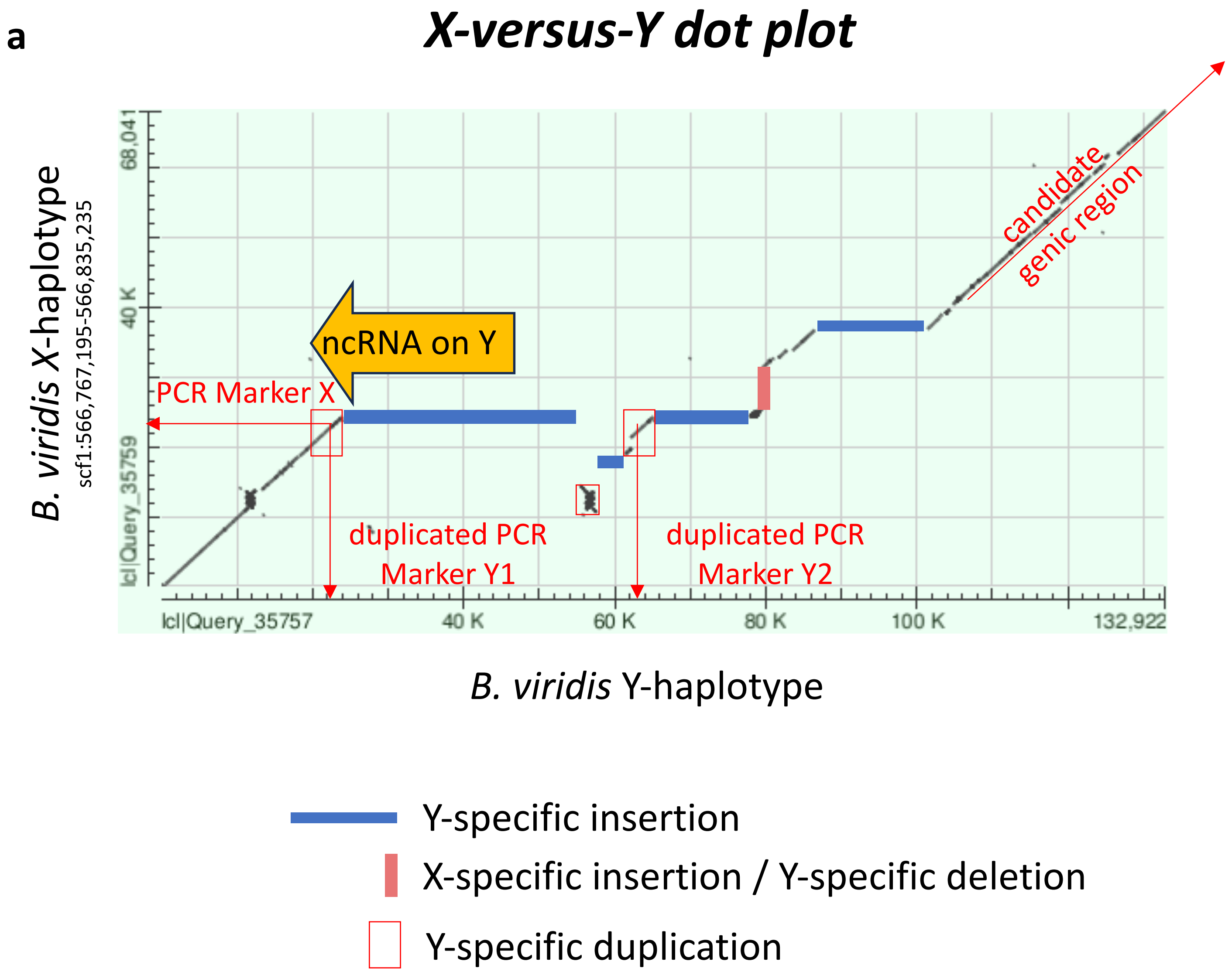

b

Y-versus-Y dot plot

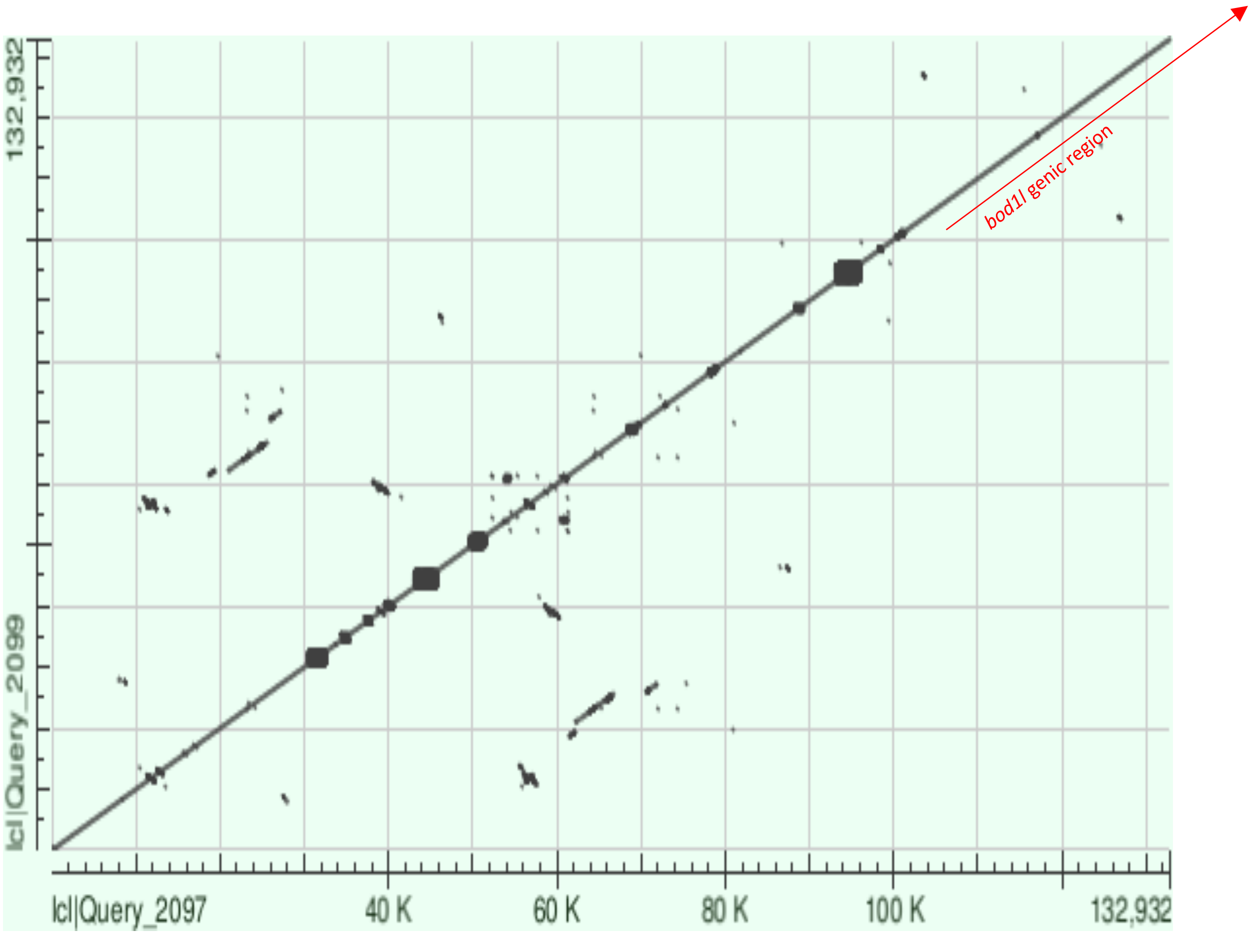

B. viridis Y-haplotype

B. viridis Y-haplotype

c

X-versus-X dot plot

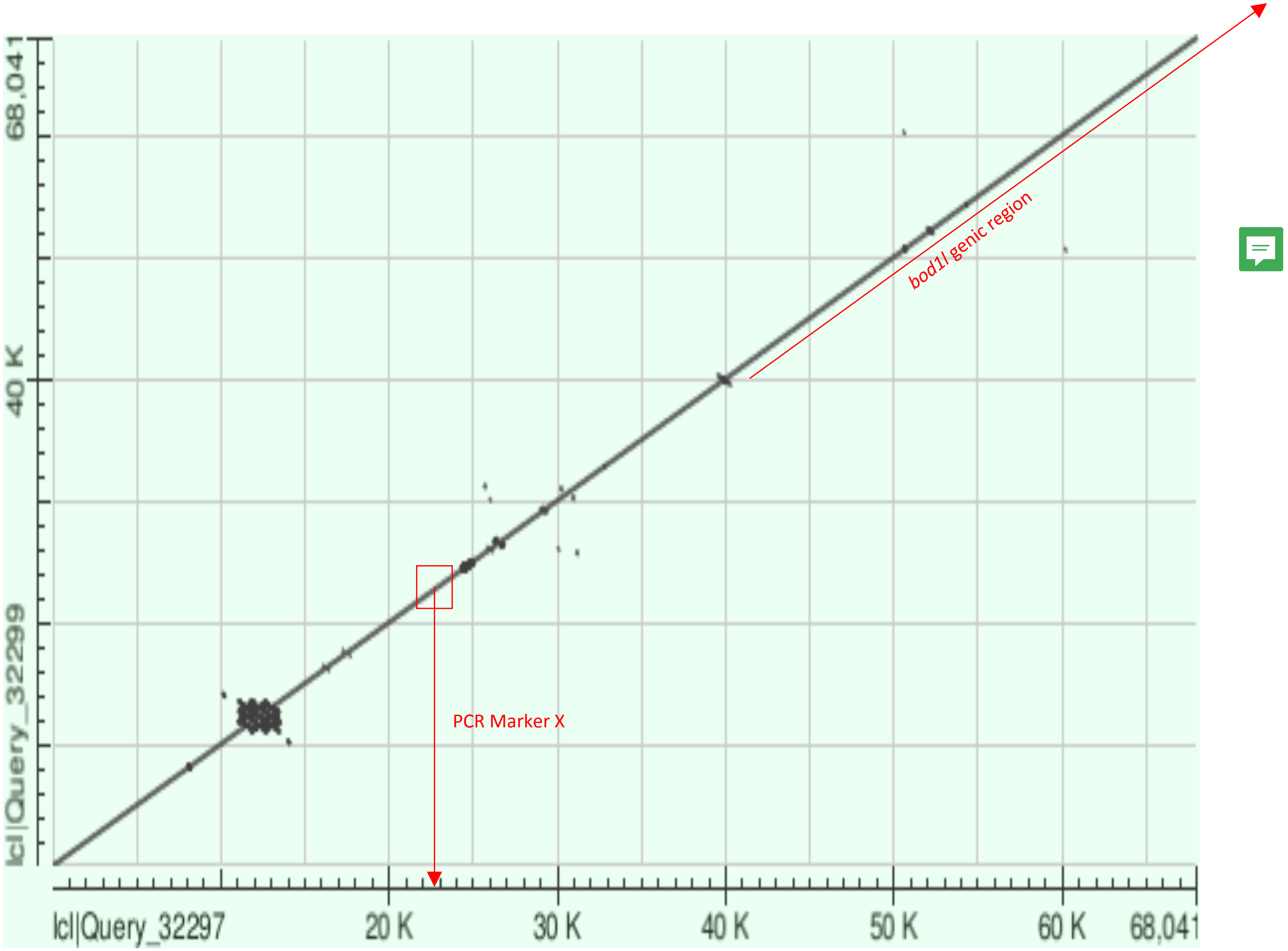

B. viridis X-haplotype
scf1:566,767,195-566,835,235

B. viridis X-haplotype
scf1:566,767,195-566,835,235

Supplementary Figure 8

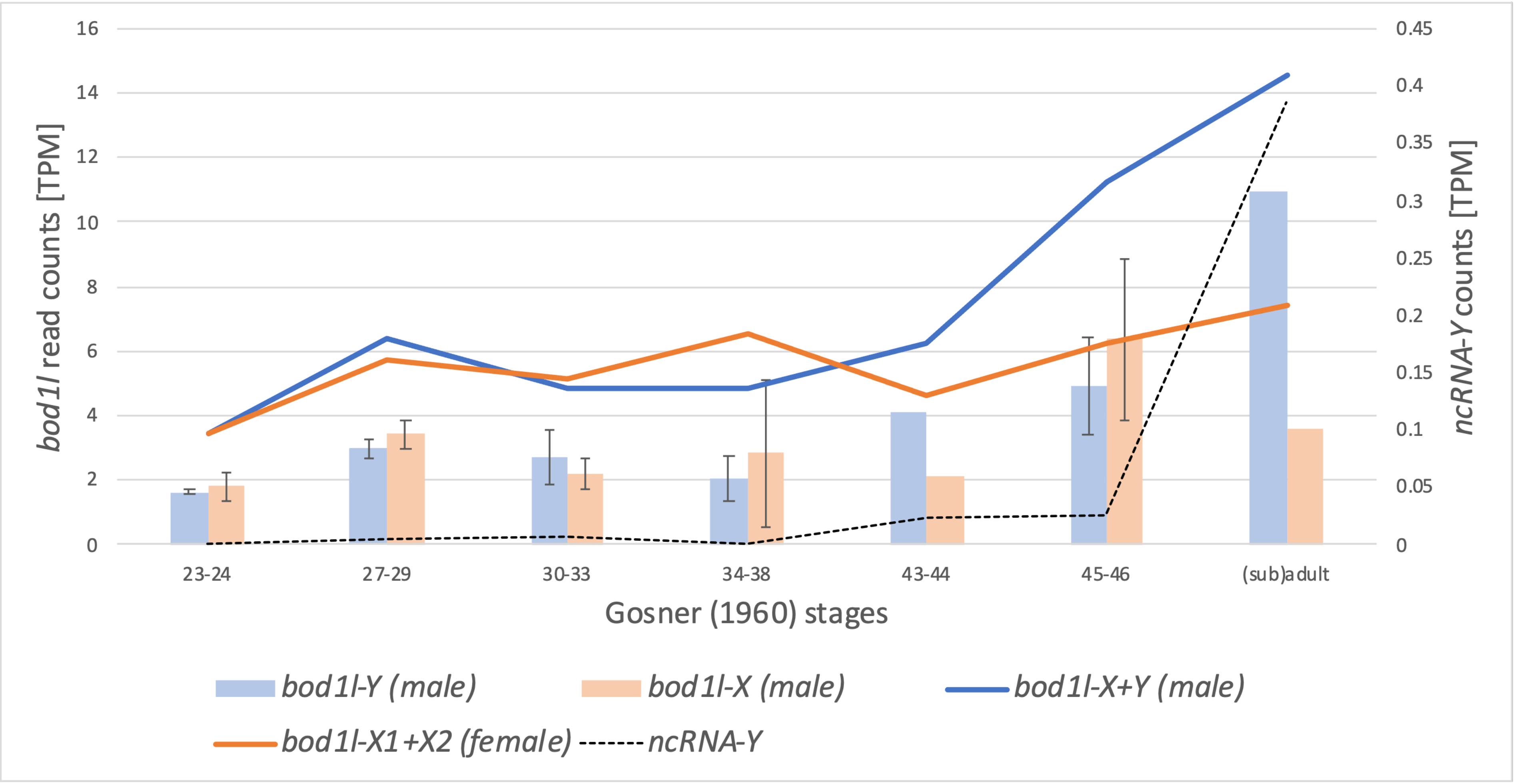

Supplementary Figure 9

a

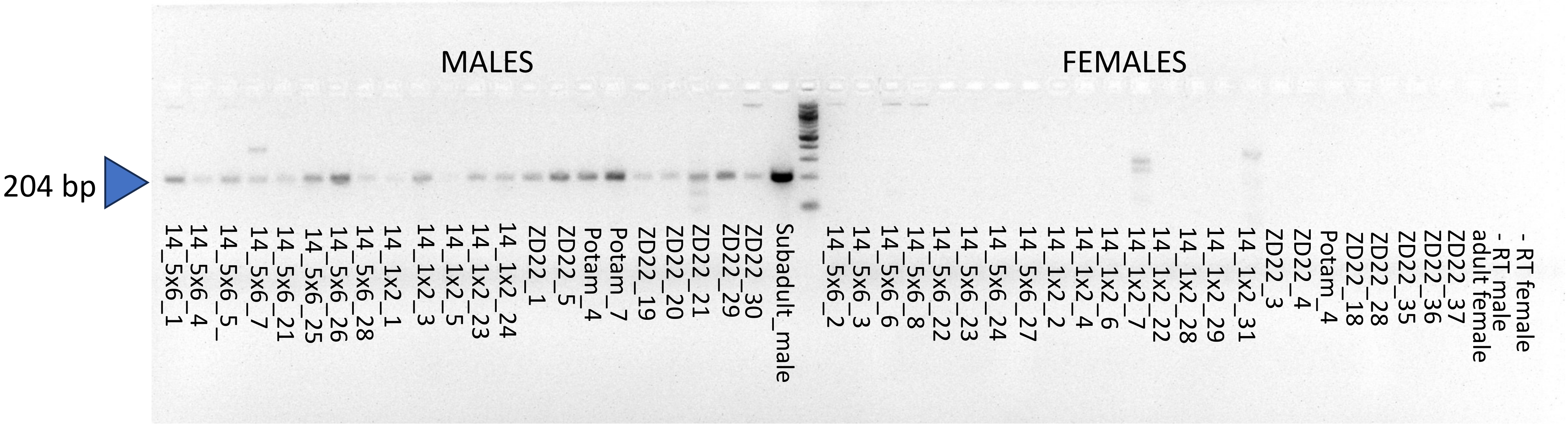

Primers: NcRNAY_Ex1to2_F + NcRNAY_Ex3to2_R

b

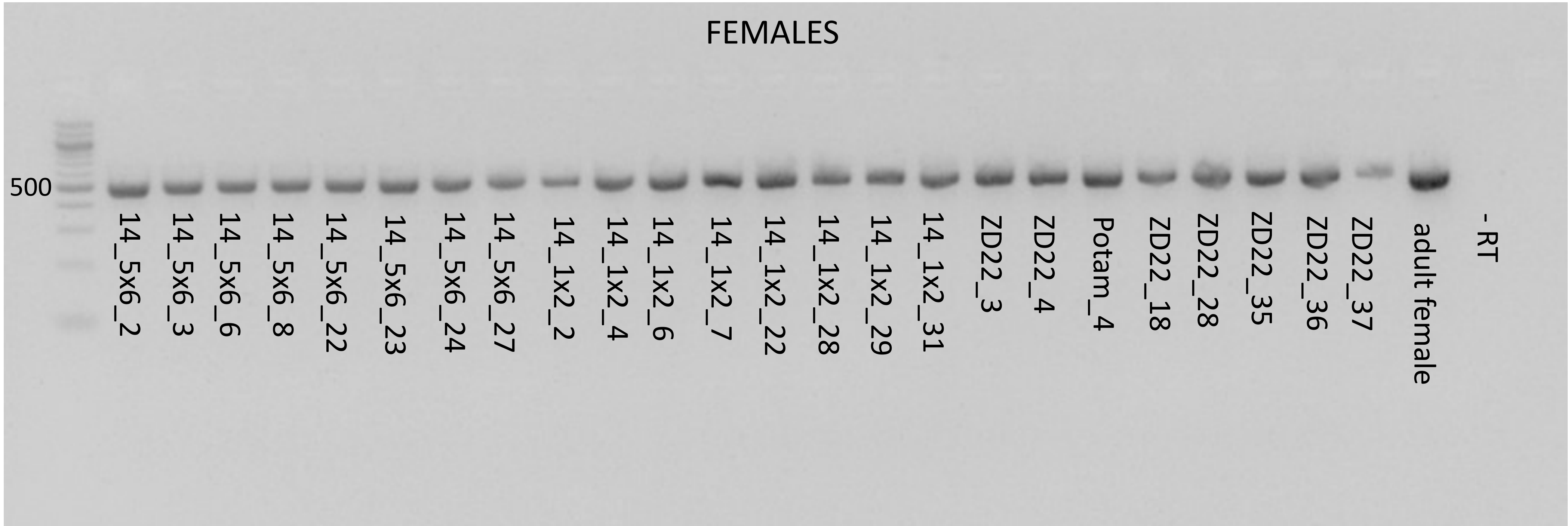

Primers: vasa_1F/R

Supplementary Figure 10

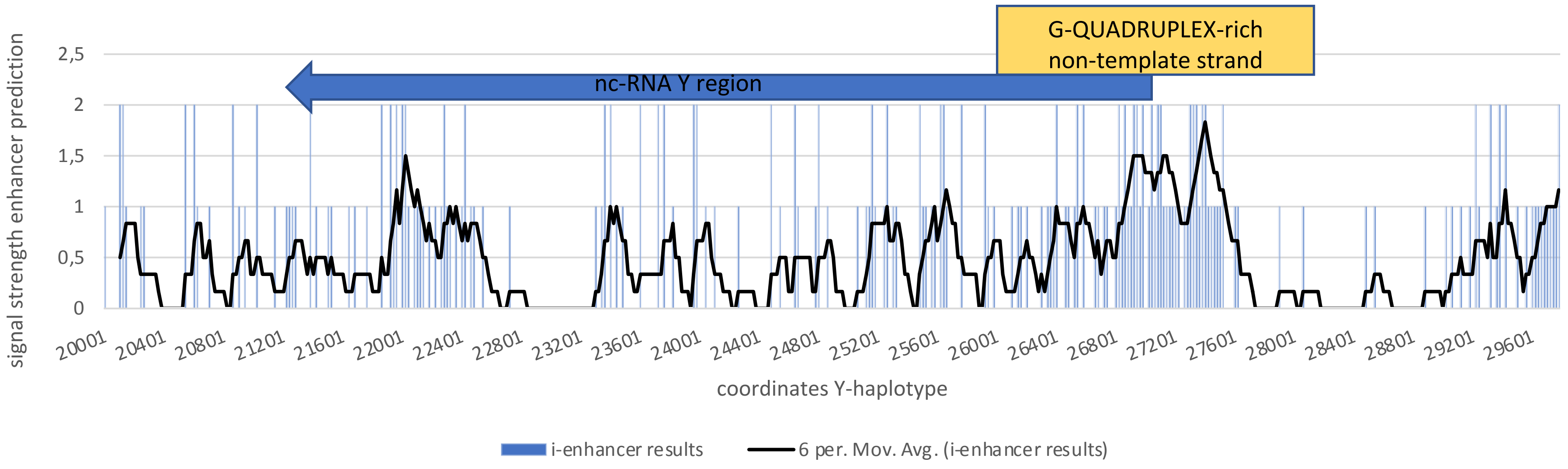

Supplementary Figure 11

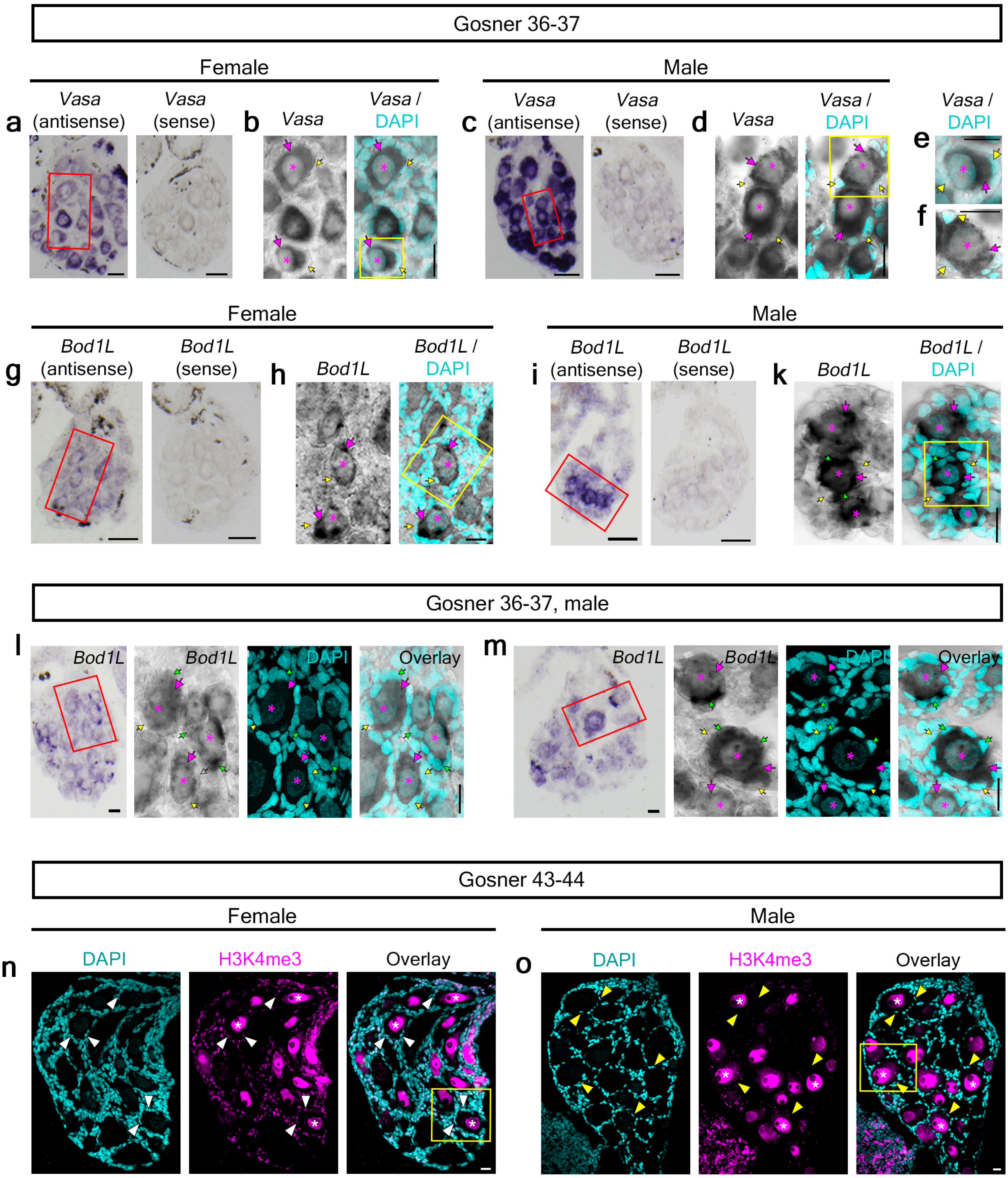

Supplementary Text 1

Annotation pipeline of protein-coding genes for the reference genome of *Bufotes viridis*

Our annotation pipeline consisted of RNAseq mapping of *Bufotes viridis* and closely related *Bufo gargarizans* to the reference genome by HiSat2 (1). Transcript models were constructed using Stringtie (2) and the mapped RNAseq reads. Additionally, closely and distantly related amphibian proteins (species and data accessions listed below) were mapped to the genome reference by SPALN (3). All resulting transcript models from both approaches were merged by Stringtie and genomic CDS locations were calculated by TransDecoder (4). Final protein predictions were aligned by Last-aligner (5) with the amphibian reference proteins (see below) to assign functions and scored by BUSCO (6).

Transcriptomes short reads (RNAseq) from *Bufotes viridis* (SRR2163277) and *Bufo gargarizans* (SRR11637930 - SRR11637935) were used.
Reference annotation proteins were included from: *Nanorana parkeri* (GCF_000935625.1_ASM93562v1_protein.faa), *Xenopus tropicalis* (GCF_000004195.4_UCB_Xtro_10.0_protein.faa), *Xenopus laevis* (GCF_001663975.1_Xenopus_laevis_v2_protein.faa), *Rhinatrema bivittatum* (GCF_901001135.1_aRhiBiv1.1_protein.faa*), Microcaecilia unicolor* (GCF_901765095.1_aMicUni1.1_protein.faa) and *Geotrypetes seraphini* (GCF_902459505.1_aGeoSer1.1_protein.faa).
Proteins used in annotation were also extracted from amphibian transcriptome assemblies from TSA by TransDecoder, using data obtained for *Bufotes viridis* (GDRL01.1.fsa_nt), *Bufo gargarizans* (assembly of SRA data above), *Pseudacris regilla* (GAEI01.1.fsa_nt), *Microhyla fissipes* (GECV01.1.fsa_nt), *Dryophytes cinereus* (GENE01.1.fsa_nt), *Rhinella marina* (GFMT01.1.fsa_nt), *Rhinella arenarum* (GHCG01.1.fsa_nt), *Oreobates cruralis* (GFNJ01.1.fsa_nt), *Anaxyrus baxteri* (GGUQ01.1.fsa_nt; GGUR01.1.fsa_nt; GGUS01.1.fsa_nt;), *Oophaga pumilio* (GIKS01.1.fsa_nt; GIKS01.2.fsa_nt) and *Boana pugnax* (GINY01.1.fsa_nt; GINY01.2.fsa_nt; GINY01.3.fsa_nt; GISC01.1.fsa_nt).

References:

1. Kim, D., Paggi, J.M., Park, C. *et al.* Graph-based genome alignment and genotyping with HISAT2 and HISAT-genotype. *Nat Biotechnol* **37**, 907–915 (2019).
2. Pertea M., Pertea GM, Antonescu CM, Chang TC, Mendell JT & Salzberg SL. StringTie enables improved reconstruction of a transcriptome from RNA-seq reads. *Nat Biotechnol* **33**, 290–295 (2015).
3. Iwata H. and Gotoh, O. Benchmarking spliced alignment programs including Spaln2, an extended version of Spaln that incorporates additional species-specific features. *Nucleic Acids* Res **40**, e161 (2012).
4. Haas, BJ. https://github.com/TransDecoder/TransDecoder .
5. Kiełbasa S.M., Wan R., Sato K., Horton P., Frith M.C. Adaptive seeds tame genomic sequence comparison. *Genome Res*. **21**, 487-493 (2019).
6. Manni M., Berkeley, M.R., Seppey M., Simão F.A., Zdobnov E.M., BUSCO Update: Novel and streamlined workflows along with broader and deeper phylogenetic coverage for scoring of eukaryotic, prokaryotic, and viral genomes. *Mol Biol Evol*, **38**, 4647–4654 (2021).

Structural comparison of the gene products from the X- and Y-copy of *bod1l*

A closer look at the SNPs in the transcriptomes (Supplementary File 5) revealed that two coding SNPs led to non-synonymous changes between the X and the Y copy, specifically scf1:566,831,998 G/A (AA609: Arginine to Lysine) and scf1:566,832,081 T/G (AA637: Serine to Alanine). To test if these amino-acid changes may cause structural and thus functional differences to explain sex-specific action of the X and Y copies of the manually curated *Bod1L* gene model, we evaluated them using the platforms Alphafold and RaptorX. Indeed, folding differences occur, but their predictions remained highly insecure due to the low quality of the Alphafold model for these extremely large proteins.

While our results in *B. viridis* could neither confirm nor fully reject that structural and thus functional differences of the *Bod1L*-proteins might play sex determining roles, together with the results of the AmpliSeq-approach from multiple related green toad species, that were not showing any conserved Y-specific coding mutations between the species, we currently consider the X- and Y-specific differences in the coding region not to be of primary importance for sex determination.
