# Supplementary material for: A candidate sex determination locus in amphibians which evolved by structural variation between X- and Y-chromosomes
Source: Nat Commun. 2024 Jun 5;15:4781. doi: 10.1038/s41467-024-49025-2 (PMC11153619; doi:10.1038/s41467-024-49025-2)
Supplement: Supplementary file 3 — Reporting Summary [file 41467_2024_49025_MOESM3_ESM.pdf]

nature portfolio

Corresponding author(s): Matthias Stöck, Christoph Winkler

Last updated by author(s): May 3, 2024

Reporting Summary

Nature Portfolio wishes to improve the reproducibility of the work that we publish. This form provides structure for consistency and transparency in reporting. For further information on Nature Portfolio policies, see our Editorial Policies and the Editorial Policy Checklist.

Statistics

For all statistical analyses, confirm that the following items are present in the figure legend, table legend, main text, or Methods section.

n/a	Confirmed	
☐	☒	The exact sample size (*n*) for each experimental group/condition, given as a discrete number and unit of measurement
☒	☐	A statement on whether measurements were taken from distinct samples or whether the same sample was measured repeatedly
☐	☒	The statistical test(s) used AND whether they are one- or two-sided<br>*Only common tests should be described solely by name; describe more complex techniques in the Methods section.*
☒	☐	A description of all covariates tested
☒	☐	A description of any assumptions or corrections, such as tests of normality and adjustment for multiple comparisons
☐	☒	A full description of the statistical parameters including central tendency (e.g. means) or other basic estimates (e.g. regression coefficient) AND variation (e.g. standard deviation) or associated estimates of uncertainty (e.g. confidence intervals)
☐	☒	For null hypothesis testing, the test statistic (e.g. *F*, *t*, *r*) with confidence intervals, effect sizes, degrees of freedom and *P* value noted<br>*Give P values as exact values whenever suitable.*
☒	☐	For Bayesian analysis, information on the choice of priors and Markov chain Monte Carlo settings
☒	☐	For hierarchical and complex designs, identification of the appropriate level for tests and full reporting of outcomes
☒	☐	Estimates of effect sizes (e.g. Cohen's *d*, Pearson's *r*), indicating how they were calculated

Our web collection on statistics for biologists contains articles on many of the points above.

Software and code

Policy information about availability of computer code

Data collection	All sequencing data has been collected using the at the time (2020-23) most recent kits , corresponding protocols and software available from Illumina or Oxford Nanopore Technologies. Novogene served as a sequencing provider.
Data analysis	IDBA-UD v1.1; WTDBG v2.2; Flye v2.9.2-b1786; bcftools v 0.1.19-96b5f2294a; Juicer v1.5; 3dDNA v180419; Juicebox v1.11.08; RepeatModeler v.1.0.8; Minimap2 v 2.26-r1175; dorado v0.5.1; PECAT v 0.0.3-0; Chromap v0.2.4-r467; Yahs v1.2a.1; RAGOUT v2.3; BUSCO v3.0.2; Stacks v2.55; RADSEX v1.2.0; Samtools v1.19.1; Awk v 5.1.0; Platypus v0.8.1.2-6; Bedtools v2.31.1; Guppy v6.0; NGSpeciesID v0.1.1.1; MAFFT v7.271; GBLOCKS v0.91b; IQtree v1.6.12; iEnhancer-Deep (website: http://nsclbio.jbnu.ac.kr/tools/iEnhancer-Deep/ accessed 08/2023); STAR aligner v2.7; SUBREAD v2.0.3; ColabFold v12/2022; HiSat2 v2.2.0; Stringtie v2.2.1; SPALN v2.0.6f; TransDecoder v5.5.0; LAST-aligner v1454; command lines and scripts https://github.com/HMPNK/BUFVIR

For manuscripts utilizing custom algorithms or software that are central to the research but not yet described in published literature, software must be made available to editors and reviewers. We strongly encourage code deposition in a community repository (e.g. GitHub). See the Nature Portfolio guidelines for submitting code & software for further information.

Data

Policy information about availability of data

All manuscripts must include a data availability statement. This statement should provide the following information, where applicable:

- Accession codes, unique identifiers, or web links for publicly available datasets
- A description of any restrictions on data availability
- For clinical datasets or third party data, please ensure that the statement adheres to our policy

All data generated in this study have been deposited in publicly accessible databases. Genome assembly, whole genome sequencing (WGS) data and RNA-seq reads are all publically accessible in the GenBank Bioproject: PRJNA292872. Furthermore, the IGB-Green Toad genome browser http://genomes.igb-berlin.de/Greentoad/ provides easy access to the assembly and its annotation. GenBank accession numbers used for genome annotation are listed in the Supplementary Text.
Code availability
Scripts and parameters are deposited at https://github.com/HMPNK/BUFVIR.

Research involving human participants, their data, or biological material

Policy information about studies with human participants or human data. See also policy information about sex, gender (identity/presentation), and sexual orientation and race, ethnicity and racism.

Reporting on sex and gender	N.A.
Reporting on race, ethnicity, or other socially relevant groupings	N.A.
Population characteristics	N.A.
Recruitment	N.A.
Ethics oversight	N.A.

Note that full information on the approval of the study protocol must also be provided in the manuscript.

Field-specific reporting

Please select the one below that is the best fit for your research. If you are not sure, read the appropriate sections before making your selection.

☒ Life sciences ☐ Behavioural & social sciences ☐ Ecological, evolutionary & environmental sciences

For a reference copy of the document with all sections, see nature.com/documents/nr-reporting-summary-flat.pdf

Life sciences study design

All studies must disclose on these points even when the disclosure is negative.

Sample size	for each experiment, sample sizes are provided in the Methods section; given, we examined samples from wild-caught animals, we tried best to get the highest available sample numbers; this had also carefully to be balanced with ethical issues when animals had to be killed for obtaining data.
Data exclusions	no data were excluded
Replication	We used 3 or more green toad males and females for the 6 developmental stages for RNAseq; while not all samples could be successfully analysed, successful confriming results; detailed data are provided in the Methods section.
Randomization	In larval and juvenile stage, females and males could not be distinguished, ensuring random sampling until the molecular marker revealed the genetic sex; for adults, application of phenotypic sexing is described in the Methods section
Blinding	Blinding was not possible since we compared phenotypic with genetic sex or DNA-based sex markers to sex-specific gene expression

Reporting for specific materials, systems and methods

We require information from authors about some types of materials, experimental systems and methods used in many studies. Here, indicate whether each material, system or method listed is relevant to your study. If you are not sure if a list item applies to your research, read the appropriate section before selecting a response.

Materials & experimental systems

n/a	Involved in the study
☐	☒ Antibodies
☒	☐ Eukaryotic cell lines
☒	☐ Palaeontology and archaeology
☐	☒ Animals and other organisms
☒	☐ Clinical data
☒	☐ Dual use research of concern
☒	☐ Plants

Methods

n/a	Involved in the study
☒	☐ ChIP-seq
☒	☐ Flow cytometry
☒	☐ MRI-based neuroimaging

Antibodies

Antibodies used

Rabbit polyclonal anti-histone H3 (tri methyl K4) primary antibody 1:100; (Abcam ab8580) and secondary goat anti-rabbit Alexa Fluor 568 (1:500, Invitrogen A-11011) were used.

Validation

According to the manufacturers, validated to work in anurans (Xenopus);
https://www.citeab.com/antibodies/763751-ab8580-anti-histone-h3-tri-methyl-k4-antibody-chi

Animals and other research organisms

Policy information about studies involving animals; ARRIVE guidelines recommended for reporting animal research, and Sex and Gender in Research

Laboratory animals

No laboratory animals were used in the study.

Wild animals

Most adult toads were sampled more than 20 years ago by either minimal invasive sampling methods using tail tips of larvae or saliva of adults obtained by buccal cotton swabs. These animals had been released after sampling. We also used tissues of road kill toads and/or collected a few adult animals in Greece. See also: Field-collected samples and Ethics section.

Reporting on sex

Our paper is about genetic sex determination; details on phenotypic and molecular genetic sexing are provided in detail within the paper. Female and male animals were likewise included in our study.

Field-collected samples

For sampling and export of toads and tadpoles, permits were kindly provided by the Ministry of the Environment of Greece (115790/229) 2014-2017. Developmental stages of tadpoles were obtained from control groups of an animal experiment approved by the German State Office of Health and Social Affairs (LaGeSo, Berlin, Germany; G0359/12) and a commercial breeder.

Ethics oversight

Our research complies with all relevant ethical regulations; the relevant animal protection committee of the IGB and the city of Berlin (LAGESO) approved the study protocol where relevant. The tadpoles were kept according to approved practice (permit ZH 114 to IGB; LAGESO Berlin, Germany), including the provision of appropriate tank size, sufficient rate of waterflow, natural photoperiod, ad libitum food supply, as well as temperatures within the species' thermal tolerance range. This ensured that no pain, suffering, distress or lasting harm was inflicted on the animals. According to the permit G0359/12, tadpoles and juvenile toads were euthanized by an overdose of Tricaine PHARMAQ 1000mg/g (MS222) since this does not incur pain, suffering or distress.

Note that full information on the approval of the study protocol must also be provided in the manuscript.

Plants

Seed stocks

N.A.

Novel plant genotypes

N.A.

Authentication

N.A.
