# Supplementary material for: A candidate sex determination locus in amphibians which evolved by structural variation between X- and Y-chromosomes
Source: Nat Commun. 2024 Jun 5;15:4781. doi: 10.1038/s41467-024-49025-2 (PMC11153619; doi:10.1038/s41467-024-49025-2)
Supplement: Supplementary file 4 — Description of Additional Supplementary Files [file 41467_2024_49025_MOESM4_ESM.pdf]

Description of Additional Supplementary Files

File Name: Supplementary Data 1

Description: Localities/populations of European green toads that contributed to the pool-seq experiment.

File Name: Supplementary Data 2

Description: Summary of the pool sequencing approach for scaffold 1 (scf1) for SNPs homozygous in males/heterozygous females (Z/W) as well as SNPs heterozygous in males/homozygous females (X/Y).

File Name: Supplementary Data 3

Description: Analyses of SNPs causing 6 non-synonymous changes in the coding region of bod1l in 49 male and female transcriptomes as compared to the female reference genome. None of these changes was 100% associated with genetic sex as in the 5'-region of bod1l.

File Name: Supplementary Data 4

Description: Percentages of covered parts of ca. 80 kb of the bod1l-region by targeted sequencing in six diploid green toad taxa, using a panel based on the female B. viridis reference genome, compared to ca. 20 kb of the gene for *anti-Muellerian hormone* (*amh*). Taxa abbreviations: Bbal - *balearicus*, Bsha - *shaartusiensis*, Bsic - *siculus*; Btur - *turanensis*, Bvir - *viridis* and *variabilis.*

File Name: Supplementary Data 5

Description: SNP-comparisons in ca. 80 kb of the bod1l- (scf1: 566,783,513 - 566,865,063) and ca. 10 kb of the amh-regions (scf1: 324,181,734 – 324,192,327) by targeted sequencing in six diploid green toad taxa in a panel based on the female *B. viridis* reference genome. The coordinates of the cluster of male specific SNPs present in all five species are colored in red (scf1: 566,790,085 - 566,790,204). Values for colorcoded SNP-comparisons with the reference genome: >=0.9: homozygous (green/yellow) = identical to reference; between 0.9 and 0.1: heterozygous (orange) = one allele identical to reference; <=0.1: homozygous(red) = completely different from reference; NA: SNP not covered by sequencing data in the relevant individual / taxon; M - male, F - female; position: SNP position on the female *B. viridis* reference genome; taxa abbreviations as in Supplementary Data 3.

File Name: Supplementary Data 6

Description: Comparison of female and male B. viridis genome assemblies.

File Name: Supplementary Data 7

Description: : Boxplots showing expression of 80 genes that play roles in sexual development and/or sex determination in other vertebrates (also known as ‘usual suspects’1 , in developmental stages of *B. viridis*. X-axis showing the normalized intensity of expression; Y-axis marks six developmental stages (details as in Fig. 3).

File Name: Supplementary Data 8

Description: Signal of the AI-based software i-enhancer, reaching from 0 to 2, predicting strong enhancer properties of the Y-specific 5’-end of bod1l, including a G-quadruplex-rich region on the non-template strand, overlapping with the ncRNA-Y

File Name: Supplementary Data 9

Description: File of splice site read counts in *bod1l*-region
