# Supplementary material for: A candidate sex determination locus in amphibians which evolved by structural variation between X- and Y-chromosomes
Source: Nat Commun. 2024 Jun 5;15:4781. doi: 10.1038/s41467-024-49025-2 (PMC11153619; doi:10.1038/s41467-024-49025-2)
Supplement: Supplementary file 5 — Supplementary Data 1–9 [file 41467_2024_49025_MOESM5_ESM.zip › BvSex_SupplData/SupplData_6_female-male-assembly-comparison.pdf]

An additional male genome assembly of *Bufotes viridis*

- Male ONT data, which was used to assemble the Y-haplotype manually, was used for whole genome assembly together with male Hi-C data.

- Newest correction algorithms were applied to improve ONT data quality (Dorado SUP basecalling + PECAT haplotype aware read correction)

- A collapsed assembly was computed and polished using male poolseq data

- This male assembly had the Y-haplotype assembled into chr1/scf1 in front of *bod1l*. Confirming the outcome of our manual approach.

Statististics *B. viridis* male *vs*. female assemblies

Female *B. viridis* assembly:

Total: 3,796,953,604 bp
Count: 13,339
Scf N50: 470,251,507 bp
Ctg N50: 1,872,430 bp

placed in chr:
3,538,486,263 bp

Male *B. viridis* assembly:

Total: 3,660,185,173 bp
Count: 10,810
Scf N50: 464,180,742 bp
Ctg N50: 466,553 bp

placed in chr:
3,491,360,402 bp

BUSCO vertebrata odb9 on annotated genes

FEMALE: C:96.4%[S:94.2%,D:2.2%],F:1.9%,M:1.7%,n:2586
MALE: C:96.7%[S:94.3%,D:2.4%],F:1.8%,M:1.5%,n:2586

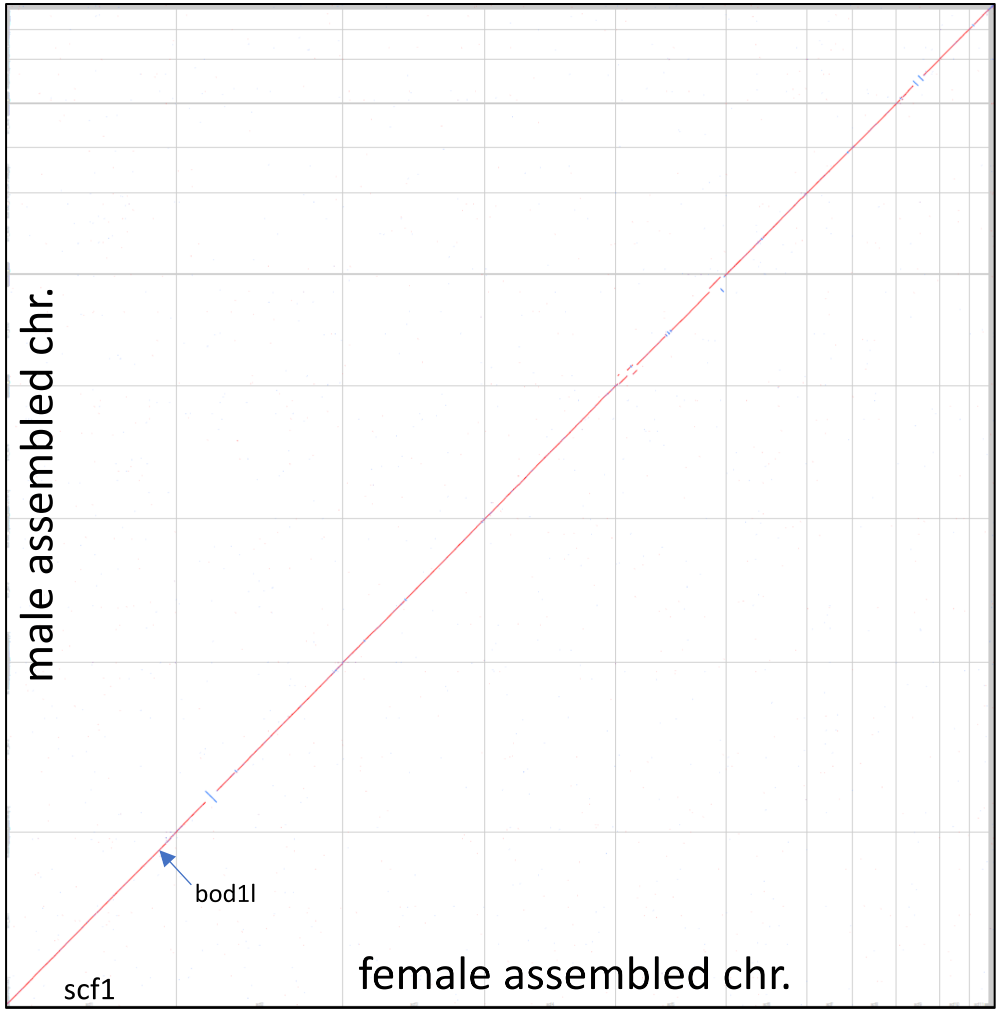

Compared to the female assembly, the male assembly has lower contig continuity and thus is a little smaller, as more repeat sequences are not resolved, but BUSCO analysis shows similar results (male assembly even slightly better) and the overall collinearity between assembled chromosomes is nearly perfect (some differences occur but are considered typical Hi-C ambiguities)

Comparison of X- and Y-haplotype assemblies (manually or automated)

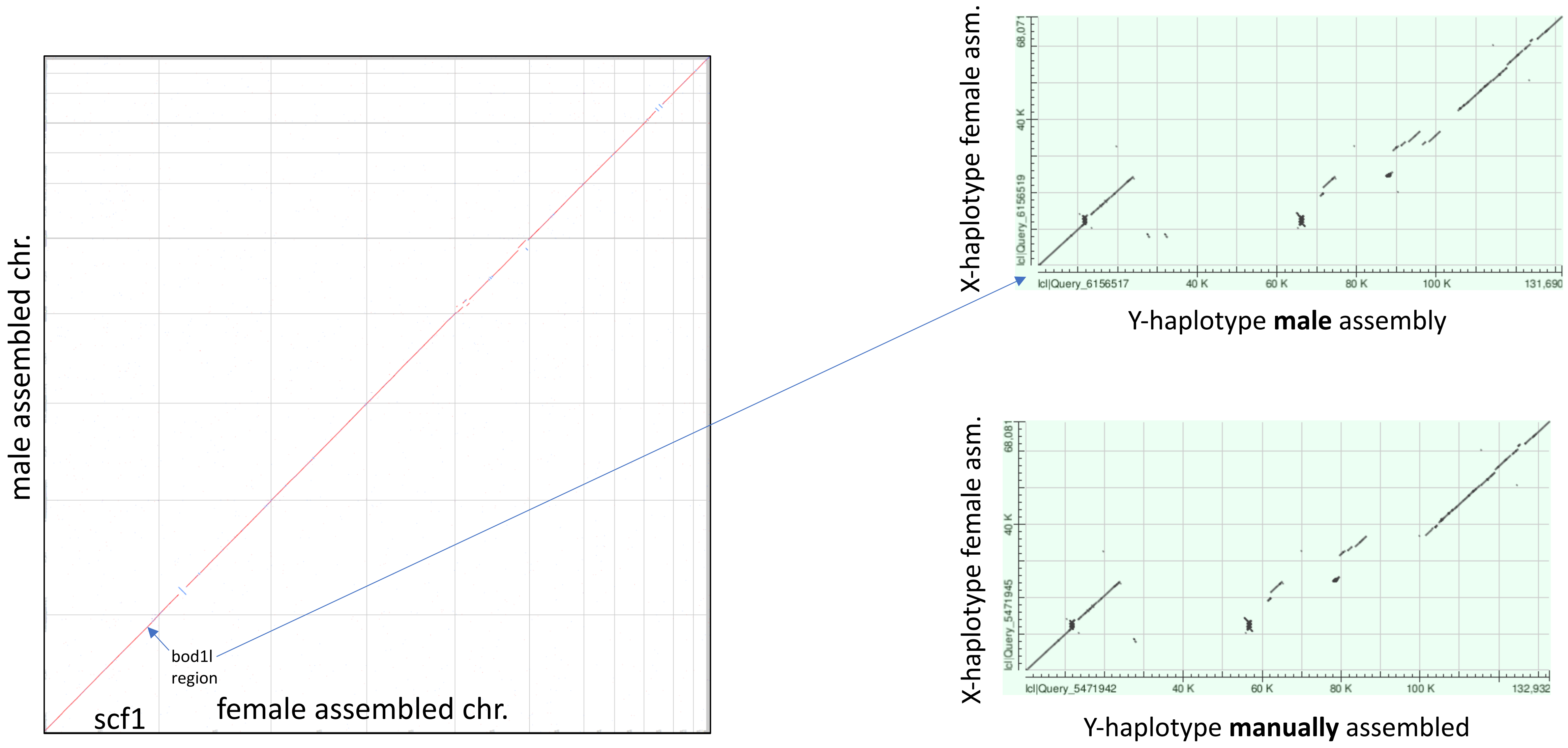

Comparison of Y-haplotype assemblies (manually *vs*. automated)

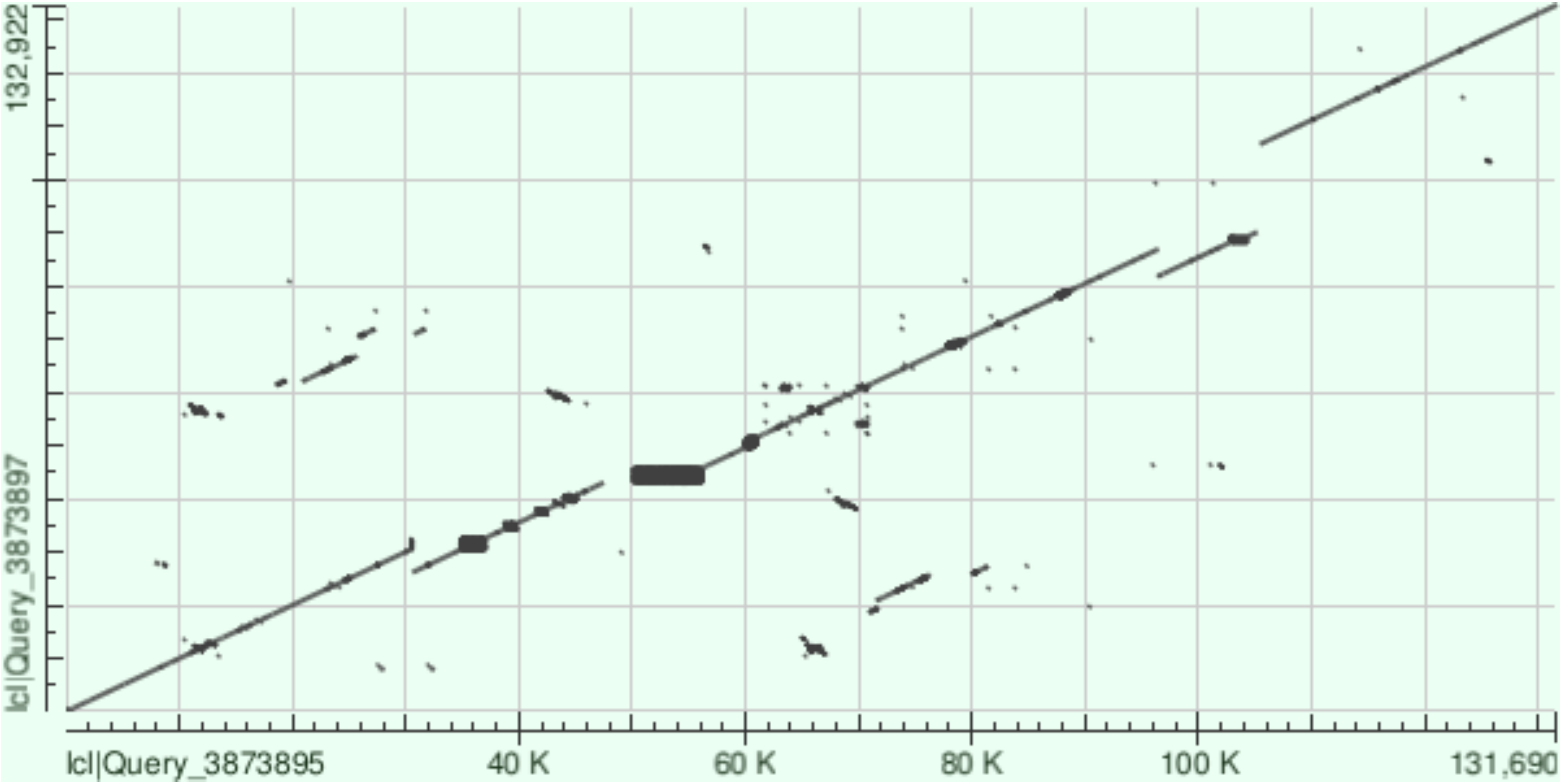

Y-haplotype **manually** assembled

Y-haplotype **male** assembly

Male/Female poolseq Y specific coverage and heterozygous SNP analysis of the <u>new male assembly</u>

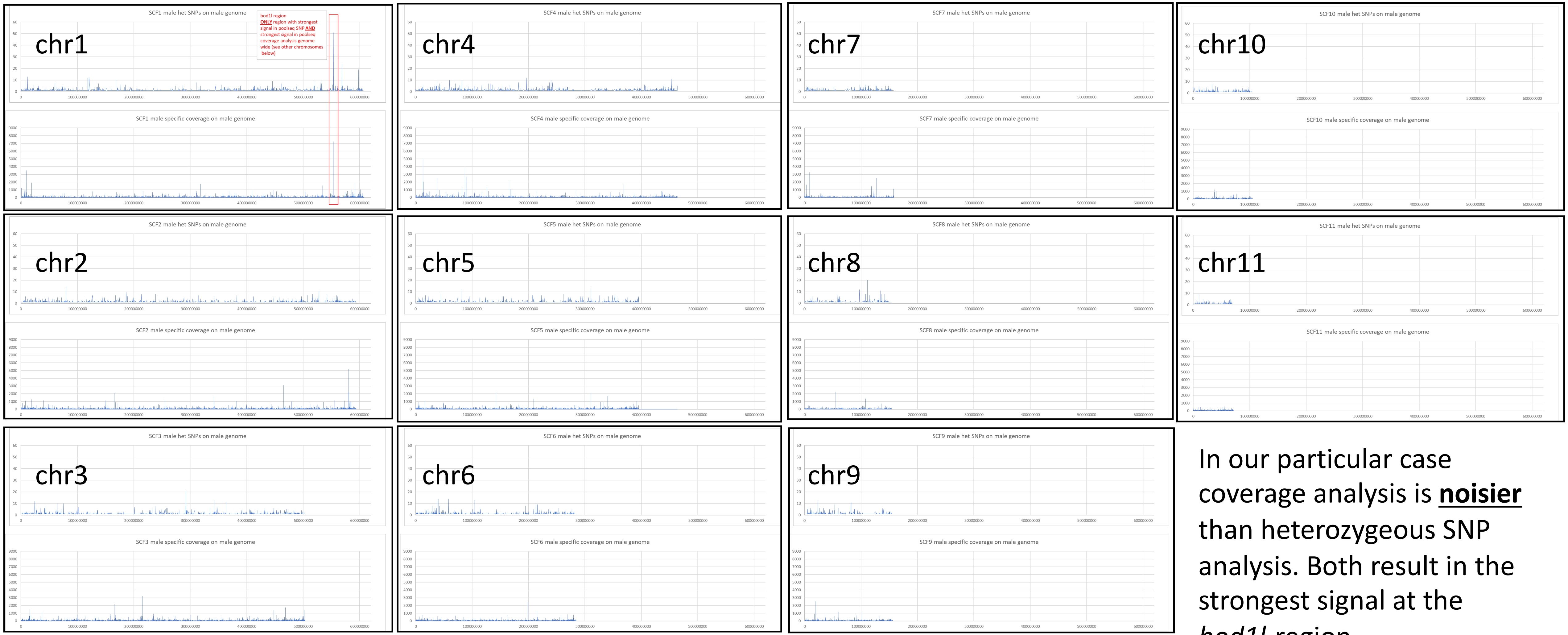

In our particular case coverage analysis is <u>**noisier**</u> than heterozygeous SNP analysis. Both result in the strongest signal at the *bod1l*-region.
The congruence of both methods at this locus is unique genome wide.

<u>**Additional test**</u>:
The top 200 (signal >700) highest scoring Y-specific poolseq coverage regions had <u>**NO**</u> overlap with any annotated „usual suspect“ sex determining gene, most of them are repeat element derived.

Male/Female poolseq het. SNP analysis on the female assembly for comparison

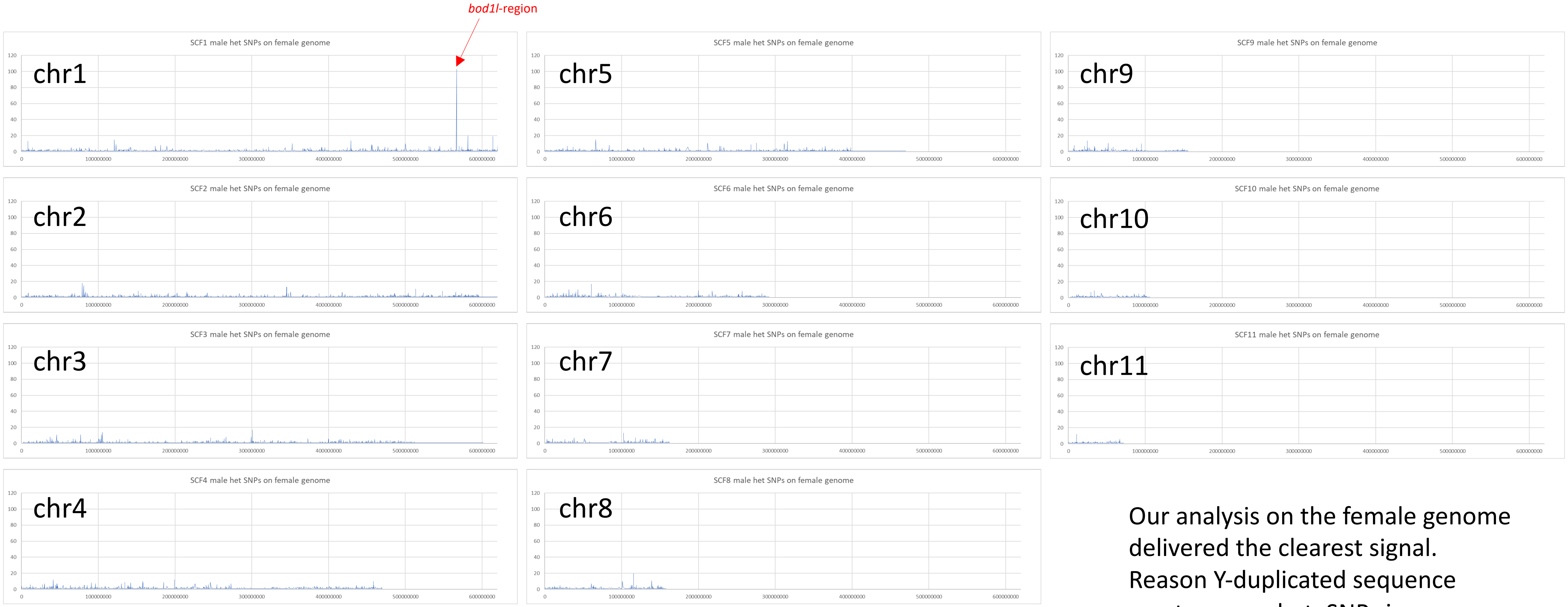

Our analysis on the female genome delivered the clearest signal. Reason Y-duplicated sequence creates more het. SNPs in non-duplicated X-reference
