# Supplementary material for: A candidate sex determination locus in amphibians which evolved by structural variation between X- and Y-chromosomes
Source: Nat Commun. 2024 Jun 5;15:4781. doi: 10.1038/s41467-024-49025-2 (PMC11153619; doi:10.1038/s41467-024-49025-2)
Supplement: Supplementary file 5 — Supplementary Data 1–9 [file 41467_2024_49025_MOESM5_ESM.zip › BvSex_SupplData/SupplData_3.pdf]

Supplementary File S3

sample no.	1	2	3	4	5	6	7	8	9	10	11	12	13	14	15	16	17	18	19	20	21	22	23	24	25	26	27	28	29	30	31	32	33	34	35	36	37	38	39	40	41	42	43	44	45	46	47	48	49
sex	F	F	F	F	F	F	F	F	F	F	F	F	F	F	F	F	F	F	F	F	F	F	F	F	F	F	F	F	M	M	M	M	M	M	M	M	M	M	M	M	M	M	M	M	M	M	M	M	M
scf1_566,831,998_G_A [AA609: Arginine to Lysine]																																																	
scf1_566,832,081_T_G [AA637: Serine to Alanine]																																																	
scf1_566,835,326_A_G [AA1718: Lysine to Glutamic acid]																																																	
scf1_566,835,600_G_T [AA1810: Alanine to Serine]																																																	
scf1_566,836,519_C_T [AA2116: Serine to Phenylalanine]																																																	
scf1_566,836,636_A_G [AA2155: Histidine to Arginine]																																																	

homozygous reference | heterozygous | homozygous alternative

sample no.	sample name short	sample name full
1	F_10_GR14_5x6-1:ref	F_10_GR14_5x6.1,/S32_1/S32_2
2	F_10_GR14_5x6-2:ref	F_10_GR14_5x6.2,/S33_1/S33_2
3	F_10_GR14_5x6-3:ref	F_10_GR14_5x6.3,/S36_1/S36_2
4	F_10_GR14_5x6-4:ref	F_10_GR14_5x6.4,/S38_1/S38_2.fq.gz
5	F_15_GR14_5x6-1:ref	F_15_GR14_5x6.1,/S40_1/S40_2.
6	F_15_GR14_5x6-2:ref	F_15_GR14_5x6.2,/S41_1/S41_2
7	F_15_GR14_5x6-3:ref	F_15_GR14_5x6.3,/S42_1/S42_2
8	F_15_GR14_5x6-4:ref	F_15_GR14_5x6.4,/S45_1/S45_2.
9	F_adult_ADULT-1:ref	F_adult_ADULT.1,/S30_1/S30_2.
10	F_ca18_GR14_1x2-1:ref	F_ca18_GR14_1x2.1,/S2_1/S2_2.
11	F_ca18_GR14_1x2-2:ref	F_ca18_GR14_1x2.2,/S4_1/S4_2
12	F_ca18_GR14_1x2-3:ref	F_ca18_GR14_1x2.3,/S6_1/S6_2
13	F_ca18_GR14_1x2-4:ref	F_ca18_GR14_1x2.4,/S7_1/S7_2
14	F_ca34_GR14_1x2-1:ref	F_ca34_GR14_1x2.1,/S13_1/S13_2
15	F_ca34_GR14_1x2-2:ref	F_ca34_GR14_1x2.2,/S14_1/S14_2
16	F_ca34_GR14_1x2-3:ref	F_ca34_GR14_1x2.3,/S12_1/S12_2
17	F_ca34_GR14_1x2-4:ref	F_ca34_GR14_1x2.4,/S9_1/S9_2.
18	F_Gosner30_Z_2022-1:ref	F_Gosner30_Z_2022.1,/S22_1/S22_2
19	F_Gosner30_Z_2022-2:ref	F_Gosner30_Z_2022.2,/S23_1/S23_2
20	F_Gosner36-37_Z_2022-1:ref	F_Gosner36-37_Z_2022.1,/S25_1/S25_2
21	F_Gosner38_Potamies-1:ref	F_Gosner38_Potamies.1,/S15_1/S15_2
22	F_Gosner38_Potamies-2:ref	F_Gosner38_Potamies.2,/S16_1/S16_2
23	F_Gosner38_Potamies-3:ref	F_Gosner38_Potamies.3,/S19_1/S19_2
24	F_Gosner38_Potamies-4:ref	F_Gosner38_Potamies.4,/S20_1/S20_2
25	F_Gosner43-44_Z_2022-1:ref	F_Gosner43-44_Z_2022.1,/S47_1/S47_2
26	F_Gosner43-44_Z_2022-2:ref	F_Gosner43-44_Z_2022.2,/S50_1/S50
27	F_Gosner43-44_Z_2022-3:ref	F_Gosner43-44_Z_2022.3,/S51_1/S51_2
28	F_Gosner43-44_Z_2022-4:ref	F_Gosner43-44_Z_2022.4,/S52_1/S52_2

sample no.	sample name short	sample name full
29	M_10_GR14_5x6-1:ref	M_10_GR14_5x6.1,/S31_1/S31_
30	M_10_GR14_5x6-2:ref	M_10_GR14_5x6.2,/S35_1/S35_2
31	M_10_GR14_5x6-3:ref	M_10_GR14_5x6.3,/S37_1/S37_2
32	M_15_GR14_5x6-1:ref	M_15_GR14_5x6.1,/S39_1/S39_2
33	M_15_GR14_5x6-2:ref	M_15_GR14_5x6.2,/S46_1/S46_2
34	M_ca18_GR14_1x2-1:ref	M_ca18_GR14_1x2.1,/S1_1/S1_2
35	M_ca18_GR14_1x2-2:ref	M_ca18_GR14_1x2.2,/S3_1/S3_2
36	M_ca18_GR14_1x2-3:ref	M_ca18_GR14_1x2.3,/S5_1/S5_2
37	M_ca18_GR14_1x2-4:ref	M_ca18_GR14_1x2.4,/S8_1/S8_2
38	M_ca34_GR14_1x2-1:ref	M_ca34_GR14_1x2.1,/S10_1/S10_2
39	M_ca34_GR14_1x2-2:ref	M_ca34_GR14_1x2.2,/S11_1/S11_2
40	M_Gosner30_Z_2022-1:ref	M_Gosner30_Z_2022.1,/S21_1/S21_2
41	M_Gosner30_Z_2022-2:ref	M_Gosner30_Z_2022.2,/S24_1/S24_2
42	M_Gosner36-37_Z_2022-1:ref	M_Gosner36-37_Z_2022.1,/S26_1/S26_2
43	M_Gosner36-37_Z_2022-2:ref	M_Gosner36-37_Z_2022.2,/S27_1/S27_2
44	M_Gosner36-37_Z_2022-3:ref	M_Gosner36-37_Z_2022.3,/S28_1/S28_2
45	M_Gosner38_Potamies-1:ref	M_Gosner38_Potamies.1,/S17_1/S17_2
46	M_Gosner38_Potamies-2:ref	M_Gosner38_Potamies.2,/S18_1/S18_2
47	M_Gosner43-44_Z_2022-1:ref	M_Gosner43-44_Z_2022.1,/S48_1/S48_2
48	M_Gosner43-44_Z_2022-2:ref	M_Gosner43-44_Z_2022.2,/S49_1/S49_2
49	M_subadult_ADULT-1:ref	M_subadult_ADULT.1,/S29_1/S29_2
