# Supplementary figures and images for: A candidate sex determination locus in amphibians which evolved by structural variation between X- and Y-chromosomes
Source: Nat Commun. 2024 Jun 5;15:4781. doi: 10.1038/s41467-024-49025-2 (PMC11153619; doi:10.1038/s41467-024-49025-2)

## Slide 1
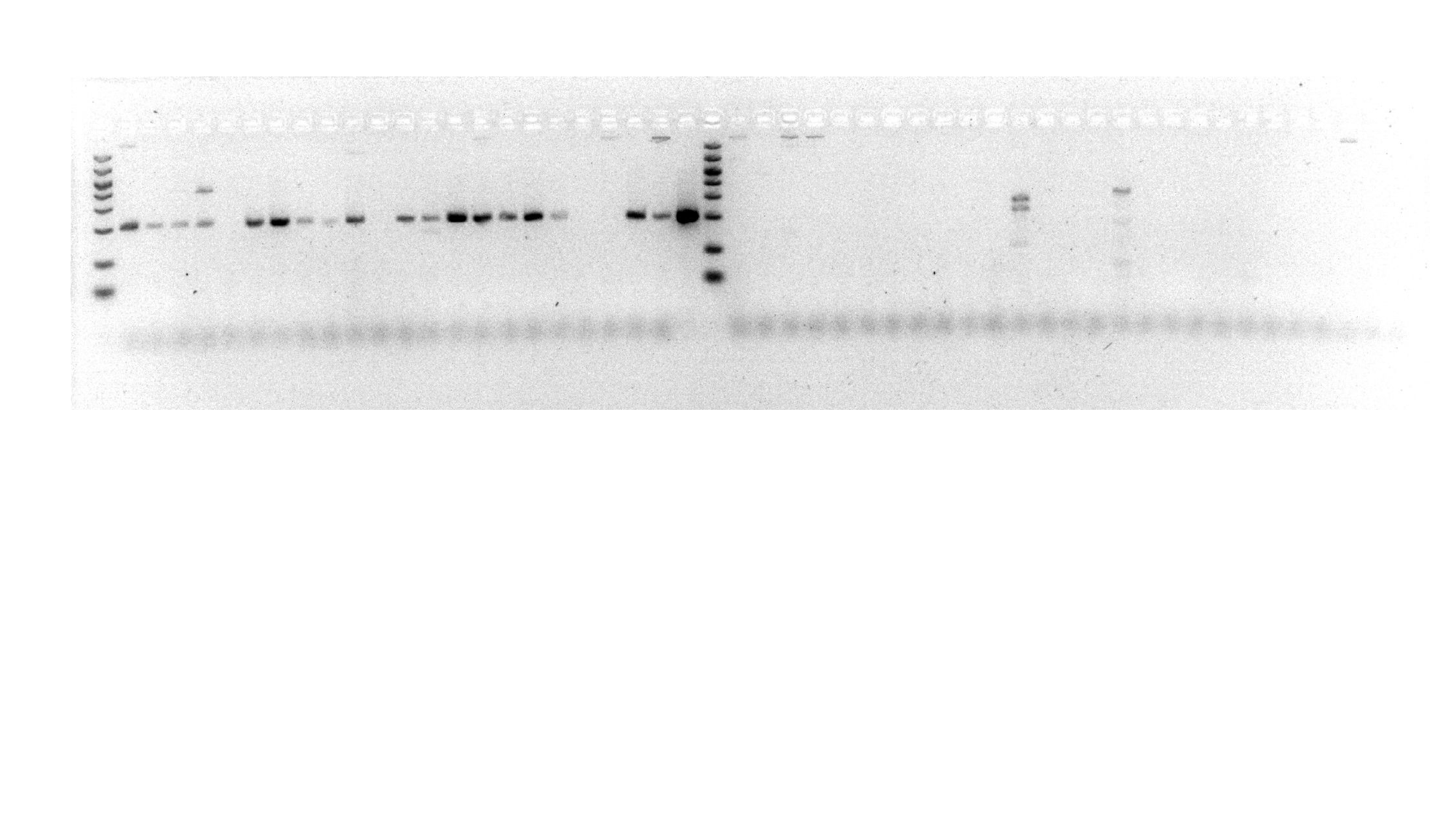

Supplement: Supplementary file 6 — Source data [file 41467_2024_49025_MOESM6_ESM.zip › source-data/source-data-Fig4c_uncropped.pptx]

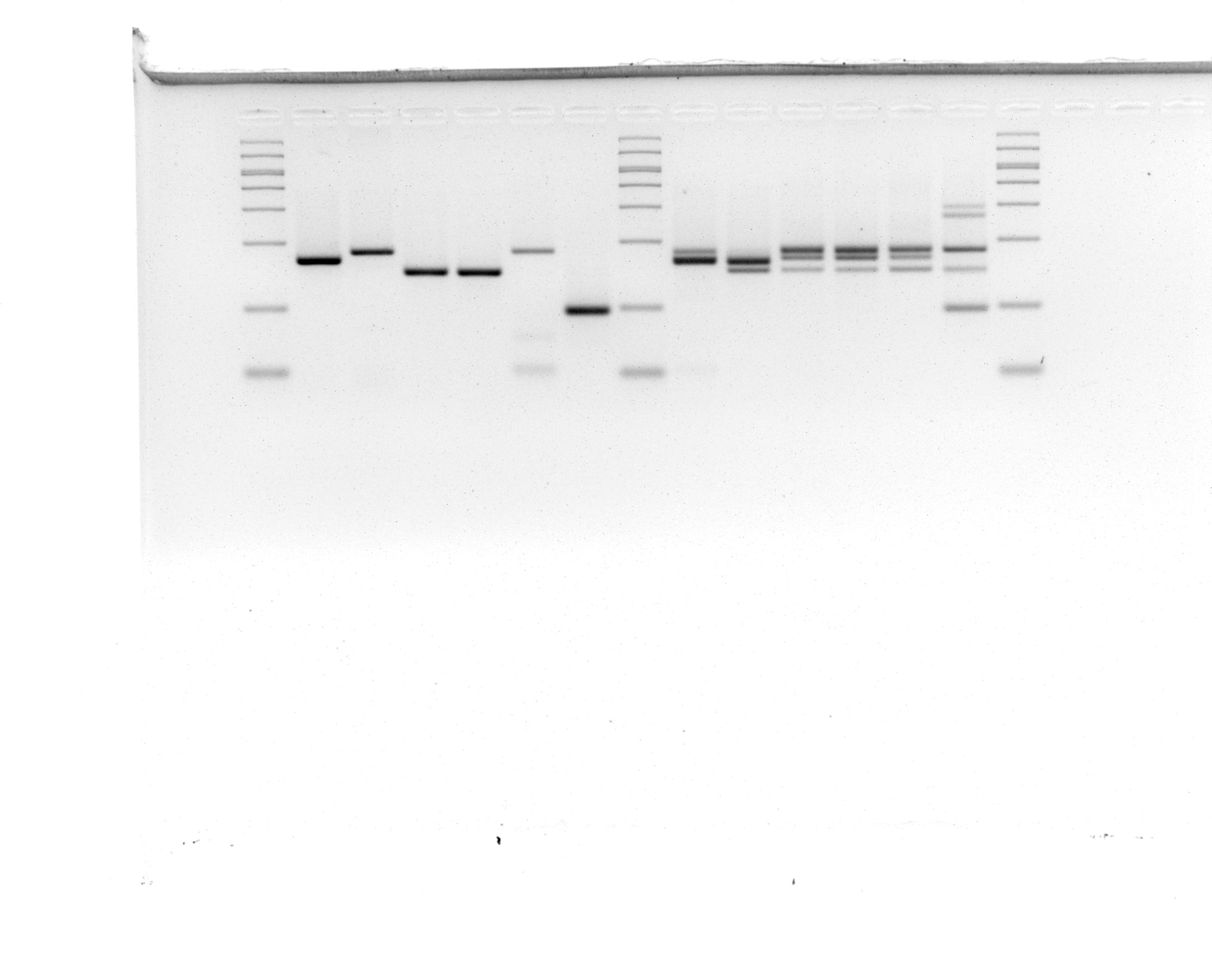

Supplement: Supplementary file 6 — Source data [file 41467_2024_49025_MOESM6_ESM.zip › source-data/source-data-Figure2b_uncropped.jpg]
